# Investigating the efficacy of uncrosslinked porcine collagen coated vascular grafts for neointima formation and endothelialization

**DOI:** 10.3389/fbioe.2024.1418259

**Published:** 2024-11-20

**Authors:** Chao Yang, Chao Su, Jie Zou, Binru Zhong, Lin Wang, Bailang Chen, Jianmo Li, Minxin Wei

**Affiliations:** ^1^ Research and Development (R&D) Department, Konee Biomedical (Shenzhen) Co., Ltd., Shenzhen, Guangdong, China; ^2^ Division of Cardiovascular Surgery, Cardiac and Vascular Center, The University of Hong Kong-Shenzhen Hospital, Shenzhen, Guangdong, China

**Keywords:** uncrosslinked collagen, coating, vascular graft, neointima, endothelization, endothelial cells, smooth muscle cell

## Abstract

**Introduction:**

This study evaluates the efficacy of uncrosslinked porcine collagen coated vascular grafts (UPCCVG) in facilitating neointima formation and endothelialization.

**Methods:**

Prior to coating, the uncrosslinked porcine collagen underwent comprehensive characterization employing SDS-PAGE, image analysis, circular dichroism and immunogenicity. The PET substrate of the vascular graft was coated with collagen solution utilizing the dip-coating method. Water permeability, blood leakage resistance, radial compliance, hemolysis, cytotoxicity and cell proliferation of UPCCVG *in vitro* were studied. Subsequent *in vivo* evaluation involved the implantation of UPCCVG as a substitute for the porcine abdominal aorta. Digital subtraction angiography (DSA) was employed to evaluate UPCCVG patency post-implantation, while histology, immunohistochemistry, and scanning electron microscopy were utilized to assess neointima formation and endothelialization. The *in vivo* thrombosis of UPCCVG was analyzed simultaneously to further characterize its blood compatibility.

**Results:**

The uncrosslinked collagen demonstrated high purity, maintaining its triple helix structure and molecular weight akin to the type I bovine collagen standard substrate, indicative of preserved biological activity and low immunogenicity. UPCCVG exhibited water permeability, blood leakage resistance, radial compliance and blood compatibility comparable to commercial grafts. DSA revealed satisfactory patency of UPCCVG without evidence of stenosis or swelling at the 3-week post-implantation mark. Histological analysis illustrated well-developed neointima with appropriate thickness and controlled proliferation. Immunohistochemistry confirmed the presence of endothelial cells (VWF positive) and smooth muscle cells (α-SMA positive) within the neointima, indicating successful endothelialization. Moreover, the morphology of the neointima surface closely resembled that of the natural artery tunica intima, oriented along the direction of blood flow.

**Discussion:**

UPCCVG, composed of uncrosslinked porcine collagen, demonstrates promising potential in fostering neointima formation and endothelialization while mitigating intimal hyperplasia. This biocompatible uncrosslinked porcine collagen merits further investigation for its clinical applications in vascular reconstruction.

## 1 Introduction

Vascular diseases pose a significant and increasing global health challenge. Recent epidemiological surveys demonstrate a continual increase in both the incidence and mortality rates linked to these conditions. Coronary heart disease and peripheral artery disease stand out among these conditions, imposing a significant toll on both individuals and healthcare systems worldwide, both in terms of lives lost and economic burdens (Mensah et al., 2019; Roth et al., 2020). In response to this pressing public health crisis, vascular grafts have emerged as indispensable tools in the therapeutic armamentarium. These grafts provide life-saving interventions for various vascular pathologies, encompassing occlusive disease, aortic aneurysms, and dissections (Hu et al., 2022). The development of effective vascular grafts relies heavily on the use of biocompatible materials coated onto synthetic textile-type graft substrates, such as Polyethylene terephthalate (PET) (Gupta and Mandal, 2021). These coatings serve two primary purposes: firstly, to reduce perioperative blood leakage and seepage, and secondly, to promote the formation of neointima—a critical tissue layer resembling the natural artery’s tunica intima—and subsequent endothelialization, where endothelial cells line the luminal surface of the neointima (Evans et al., 2021). Establishing a robust neointima and achieving complete endothelialization are crucial for ensuring long-term graft patency and reducing the risk of complications, such as thrombosis and graft failure (Zhuang et al., 2021).

Currently, the predominant coating materials for vascular grafts mainly consist of crosslinked collagen sourced from bovine, gelatin, and albumin (Manju et al., 2011; Mufty et al., 2022). Collagen, a significant component of the extracellular matrix and a fundamental element of the vascular wall, is essential for supporting the growth, differentiation, and proliferation of various vascular cells, such as fibroblasts, smooth muscle cells, and endothelial cells (Copes et al., 2019; Chen et al., 2023). Gelatin, derived from collagen degradation, demonstrates notable water solubility but lacks the critical triple helix structure that imparts structural integrity to native collagen (Alipal et al., 2021). Furthermore, albumin, the most abundant protein in blood plasma, demonstrates exceptional biocompatibility due to its minimal immunogenicity (Kuten Pella et al., 2022). These grafts remain vulnerable to numerous complications, such as thrombosis, intimal hyperplasia (excessive neointimal thickening), delayed healing, bleeding, and infection, especially regarding neointima formation and endothelialization of the graft surface (Wilson et al., 2016). Satisfactory long-term outcomes have not been consistently achieved thus far.

A recent study by Elise et al. emphasized the slow biological absorption rate of collagen coatings in explanted human grafts, noting incomplete healing and a lack of robust endothelial coverage observed in many cases (Helfer et al., 2022). However, it is crucial to acknowledge the inherent limitations of this study, as all samples were obtained from patients with compromised prognoses, including factors such as infection, thrombosis, graft stenosis (narrowing), rupture, and aneurysm degeneration, which could have significantly influenced healing and graft performance.

Furthermore, there are concerns about the potential drawbacks of the crosslinking agents commonly used in collagen-based graft coatings, including glutaraldehyde, formaldehyde, and 1-ethyl-3-(3-dimethylaminopropyl) carbodiimide/N-Hydroxysuccinimide (EDC/NHS) (Gorgieva and Kokol, 2011; Adamiak and Sionkowska, 2020). These chemical crosslinking methods have been linked to cytotoxicity due to byproducts generated during the crosslinking process, requiring complex procedures for the removal of unreacted crosslinking agents post-synthesis and subsequent re-sterilization to eliminate residual toxicity. These residual crosslinking agents, integrated into the collagen matrix, are suspected to induce cytotoxic reactions in host cells, thereby impeding endothelial cell attachment, proliferation, and ultimately, complete graft surface endothelialization (Wissink et al., 2000). Moreover, the widespread use of bovine collagen in vascular graft coatings, primarily sourced from Europe and the United States, raises concerns about potential immunogenicity and zoonotic disease transmission compared to porcine collagen (Insuasti-Cruz et al., 2022).

In comparison to crosslinked collagen, uncrosslinked collagen offers a distinct advantage by eliminating the requirement for crosslinking agents, the associated crosslinking process, and subsequent clearance procedures. This lack of crosslinking elements is expected to improve biocompatibility, making uncrosslinked collagen an attractive alternative. The uncrosslinked collagen in the form of sponge or acellular matrix rather than vascular coating, as reported in the literature, also does show some advantages (Butler et al., 2010; Fernandez-Moure et al., 2017). Additionally, in regions like China and Asia, pigs are more abundant compared to cattle, providing a readily available and potentially more cost-effective source of raw material for porcine collagen production.

This study is motivated by pressing concerns and promising prospects, aiming to explore the potential of uncrosslinked porcine collagen as a novel coating material for PET-based vascular grafts. We meticulously examine the *in vitro* and *in vivo* performance of these Uncrosslinked Porcine Collagen Coated Vascular Grafts (UPCCVG), conducting comparative analyses against commercially available counterparts of the same type. By investigating the performance of UPCCVG, our aim is to contribute to the development of more biocompatible and efficacious vascular grafts, ultimately improving patient outcomes.

## 2 Materials and methods

### 2.1 Porcine collagen extraction

Uncrosslinked type I porcine collagen [Konee Biomedical (Shenzhen) Co., LTD., China] was extracted from porcine skin obtained from specific pathogen-free pigs using an enzymatic method described in the literature (El Blidi et al., 2021; Matinong et al., 2022). Initially, the skin was sectioned into strips, followed by sequential washes with isopropyl alcohol and purified water to remove surface impurities. Subsequently, the tissue was subjected to acidification, swelling, and mechanical disruption. Type I collagen was selectively extracted from the processed skin using pepsin digestion. The extracted collagen solution was then purified through multiple rounds of centrifugation and precipitation. The purified collagen was subsequently suspended in phosphate buffered saline and sterilized via filtration. This multi-step process effectively inactivates viruses and removes potential immunogens. Finally, the collagen solution was dialyzed against purified water at 4°C for 4 days using a 100 KDa molecular weight cutoff dialysis membrane (Celarts Co., Ltd.) to remove residual salts. Prior to dialysis, the dialysis membrane was pre-treated with 2% (w/v) sodium bicarbonate and 1 mmol/L EDTA (pH 8.0) for 10 min. The dialysate was changed every 24 h. The ultimate collagen product was obtained through lyophilization at −80°C, resulting in a pure collagen sponge.

### 2.2 Molecular weight and distribution characterization of porcine collagen

The molecular weight and distribution of porcine collagen were assessed using SDS-PAGE (PowerPac Basic, BIO-RAD). Initially, the collagen was dissolved in a 0.3% acetic acid solution to achieve a final concentration of 1.25 mg/mL. Subsequently, the sample solution was mixed with 5x loading buffer containing dithiothreitol at a 1:4 volume ratio. Next, the mixture was neutralized with a 1 M NaOH solution until a blue color change was observed, followed by heating at 95°C for 5 min to denature the proteins. After cooling, the samples and protein markers were loaded onto the SDS-PAGE gel for electrophoresis. The migration patterns of the collagen bands were qualitatively compared with the protein markers to estimate the molecular weight. The identical procedure was utilized to determine the molecular weight and distribution of the type I bovine collagen standard substrate (China National Institutes for Food and Drug Control) as a control.

### 2.3 Purity characterization of porcine collagen

Collagen purity (*CP*) was determined using sample SDS-PAGE and Coomassie bright blue staining before and after collagenase enzymolysis. The optical density of each molecular weight band was assessed using a Gel imager (ChemiDoc XRS+, Bio-Rad) and Image J software (Xu et al., 2017; Sorushanova et al., 2021). Collagenase acts specifically on the site between a neutral amino acid (X) and glycine in the Pro-X-Gly-Pro sequence, degrading collagen with a triple helix structure without affecting other proteins. *CP* is calculated as follows:

When B-C≠0, *CP* was calculated according to Equation 1.

$$CP=\frac{A-\left(B-C\right)}{A}\times 100\%$$ (1)

where: A is the sum of all strip optical densities of the sample before enzymatic hydrolysis; B is the sum of all strip optical densities of the sample after enzymolysis. C is the sum of the strip optical density of the collagenase sample.

When B-C = 0, *CP* was calculated according to Equation 2.

$$CP=\frac{10000-BSA\;limit\;value}{10000}\times 100\%$$ (2)

where: BSA limit value is the band density value at the limit concentration of bovine serum albumin (BSA) dyed by Coomassie bright blue that can be detected by the naked eye.

### 2.4 Secondary structure characterization of porcine collagen

The secondary structure of porcine collagen was assessed using CD spectroscopy. Briefly, 5.0 mg of collagen was weighed and dissolved in 10.0 mL of 0.5% acetic acid solution in a 10 mL volumetric flask. The solution was mixed thoroughly to achieve a final concentration of 0.5 mg/mL. Following this, the solution was centrifuged at 10,000 rpm in 5 min. The supernatant was collected for further analysis. A solution of type I collagen standard was prepared using the same method for comparison. The prepared collagen solution (0.5 mg/mL) was loaded into a quartz cuvette with a 1 mm path length. CD spectra were acquired on a CD spectrometer at 20°C. The scanning range was set from 180 nm to 260 nm with a resolution of 0.1 nm and a scan speed of 120 nm/min. A blank cuvette containing 0.5% acetic acid solution served as the reference. Both the control and test samples were measured in triplicate, and the average values were used for further analysis.

### 2.5 The process of coating the PET substrate of vascular graft with porcine collagen

Custom-made PET substrate of vascular graft was woven from PET yarn. Prior to use, the PET substrate underwent a collagen coating process. The coating solution was prepared by dissolving collagen and glycerin in an acetic acid solution. Glycerin served as a plasticizer, enhancing the flexibility of the final coating. A multi-dip coating technique was utilized to ensure uniform deposition of collagen on the PET substrate. After the coating steps, the substrate was ventilated and dried for 48 h at room temperature in a controlled ultraclean environment. To achieve uniform distribution of the coating, the PET substrate was continuously rotated during the drying process. Subsequently, a 12-hour vacuum drying step at room temperature was conducted. The coated substrates were then stored in sealed bags and sent to Shenzhen Jinpengyuan Irradiation Technology Co., Ltd. for radiation sterilization. Finally, the sterilized vascular graft samples underwent various performance evaluations.

### 2.6 Coating ratio of UPCCVG

The coating ratio of UPCCVG was determined through gravimetric analysis by comparing the weights before and after coating. The composition of the coating was further analyzed by differential extraction using purified water and collagenase solution to separate water-soluble and water-insoluble components. For comparison, commercially available vascular grafts were included: Hemashield (Hemashield platinum^®^ woven double velour vascular graft, Intervascular, Getinge Group, France) and Gelweave (Gelweave^®^ gelatin impregnated woven vascular prosthesis, Vascutek Limited, United Kingdom). Both utilize PET substrate, with Hemashield featuring a mildly crosslinked collagen/glycerin coating and Gelweave employing a heavily crosslinked gelatin/glycerin coating (Marois et al., 1997; Madaghiele et al., 2009).

### 2.7 Surface morphology characterization of UPCCVG

The PET substrate and UPCCVG surface were coated with gold and observed using SEM (Zeiss, Gemini300, 15 kV, emission current 10 μA) to analyze the impact of the coating on the wall and cross-sectional morphology of the vascular graft.

### 2.8 Water permeability test of UPCCVG

Water permeability testing was conducted according to references (ISO7198, 2016; Zhuravleva et al., 2024). Swatches of graft material were affixed to the bottom of a cylindrical tube measuring 150 cm in length and 1.5 cm in inner diameter. The tube was positioned upright and filled with water. The water that leaked through the graft material during each of the three 60-second trials was collected. The tube was continuously refilled with water during the test to maintain constant hydrostatic pressure on the material.

### 2.9 Blood leakage resistance test of UPCCVG

Due to the significant difference in surface tension between water and blood, and the limited duration of water permeability tests, we designed a fresh heparin anticoagulant porcine blood extracorporeal circulation to detect blood leakage resistance of UPCCVG over a 4 h period. In brief, the graft was connected to a silicone tube with an outer diameter matching its own inner diameter at both ends. A section of this silicone tube was integrated into the extracorporeal circuit via an extrusion pump to facilitate blood flow. The blood flow rate was set at 4 L/min and blood pressure was maintained at 120 ± 2 mmHg. The system was continuously monitored for blood leakage or seepage from UPCCVG during the 4-hour test period.

### 2.10 Radial compliance testing of UPCCVG

Radial compliance of UPCCVG and commercially collagen coated vascular grafts was tested on a vascular compliance tester (Dynatek DCT3, Dynatek Machine Inc.) with reference to YY/T0500-2021. The test conditions are as follows: purified water as test medium; Temperature 37°C ± 1°C; Pretension 500 mN in the direction of sample length; Pulse pressure 80–120 mmHg, Dynamic load frequency 60 times/min. Radial compliance (*RC*) is calculated according to Equation 3.

$$RC=\frac{R_{P2}-R_{P1}}{R_{P1}∙\left(P2-P1\right)}\times 10^{4}$$ (3)

where: *RC* is radial compliance, expressed in %/100 mmHg; *P1* and *P2* correspond to low pressure and high pressure respectively, and the unit is mmHg. *R*_*P1*_ and *R*_*P2*_ are the measured inner diameters of the vascular graft under the corresponding pressure.

### 2.11 Blood compatibility of UPCCVG

Hemolysis and thrombosis *in vivo* induced by UPCCVG were studied in reference to GB/T 16886.4-2022 (ISO 10993-4-2017). Fresh anticoagulant rabbit blood was prepared by taking 10 mL blood from the heart of healthy rabbits and adding 0.5 mL potassium oxalate solution with a mass concentration of 20 g/L. 8 mL fresh anticoagulant rabbit blood was diluted with 10 mL sodium chloride injection at a mass concentration of 9 g/L and set aside. 1.0g vascular grafts and commercially collagen coated vascular grafts were cut into 5 mm × 25 mm pieces and placed in test tubes respectively and 10 mL 0.9% sodium chloride injection was added as the sample group. 10 mL 0.9% sodium chloride injection and 10 mL distilled water were added to each tube as negative control and positive control respectively. After all test tubes were kept at 37°C water bath for 30 min, 0.2 mL diluted rabbit blood was added to each test tube. All samples were kept at 37°C water bath for 60min. The detection solution was centrifuged at 800 g for 5 min, and the supernatant was determined the absorbance at 545 nm wavelength by ultraviolet spectrophotometer. The hemolysis rate (*HR*) of the sample was calculated according to Equation 4.

$$HR=\frac{A_{s}-A_{nc}}{A_{pc}-A_{nc}}\times 100\%$$ (4)

where: *HR* is the hemolysis rate of sample; As is absorbance of sample group; Anc and Apc are absorbance of negative control and positive control. The absorbance of the negative control should not be greater than 0.03, while the absorbance of the positive control should be 0.8 ± 0.3.

The ability of UPCCVG to form thrombus *in vivo* was evaluated by analyzing platelet adhesion and thrombus formation after implantation *in vivo*. For details, see the animal experiment section.

### 2.12 MTT assay of the uncrosslinked collagen coating of UPCCVG

Cytotoxicity of the uncrosslinked collagen coating of UPCCVG was assessed by MTT assay. Firstly, mesenchymal stem cells (MSCs) were seeded in 75-flasks with DMEM growth medium supplemented with 10% fetal bovine serum, penicillin (100 U/mL) and streptomycin (100 ug/mL) at 37°C in 5% CO_2_-humified atmosphere and harvested by trypsinization when reached 90% confluency. Cells between the 6th and 9th passage were used for further MTT assay. Secondly, UPCCVG was immersed in complete DMEM medium at a ratio of 0.1 g/mL at 37°C for 24 h under sterile conditions to obtain the extracted solution. MSCs were seeded in 96-well plate with complete DMEM medium at 6,000 cells per well and incubated for 24 h until reached half confluency. Then the medium was replaced with extracted solution and further culture for another 24 h, medium with complete medium was used as control. After 24 h, the cell viability was assessed using the MTT colorimetric assay. MTT (1 mg/mL) was added to the medium (1:10) and samples were further incubated for another 4 h. The culture medium was removed and replaced with 150 uL DMSO to dissolve the dark blue crystals. The absorbance at 490 nm was measured by a microplate reader to obtain the absolute OD value. Relative OD value of each group was obtained by the division of the absolute OD value of the control group. All tests were performed in triplicate and results are expressed as mean ± standard deviation.

### 2.13 Cell proliferation assay of the uncrosslinked collagen coating of UPCCVG

The required concentration of collagen (15ug/cm^2^) was diluted with 0.02 M HCl before use. Then the tissue culture treated 96-well plate was coated with collagen type I at room temperature for 1 h. The unbound solution was removed, and the plate was washed with cool PBS three times to obtain the collagen-coated plate. Then MSCs between the 6th and 9th passage in the logarithmic growth phase were seeded on control surface (tissue culture treated) and collagen coated surface, respectively. Cells were allowed to adhere for 1 h at room temperature under sterile environment and then was moved to cell culture incubator for further culture at 37°C in 5% CO_2_-humified atmosphere. After three growth periods (0, 48, and 72 h), cell viability was assessed using the CCK8 assay. Briefly, 10% CCK-8 was added to the medium, then placed at 37°C and cultured for 4 h. The absorbance of the reacted solution at 450 nm was measured by a microplate reader. Three parallel samples in each group were detected and the results were average.

### 2.14 Evaluation of *in vivo* performance of UPCCVG

For this study, we utilized a porcine model of abdominal aortic replacement. Five adult male and female Large White pigs (65 ± 5 kg) were sourced from a licensed laboratory animal supplier. The animals were individually housed in cages at Silver Snake Medical Technology Co., Ltd. (Guangzhou, China). All procedures were conducted in accordance with the guidelines established by the Ethical Committee for Animal Experimentation at Silver Snake Medical Co., Ltd. The pigs received standard laboratory chow, and their cages were cleaned and disinfected daily. Each pig underwent the implantation of a 5 cm vascular graft into the abdominal aorta. Graft patency was assessed using DSA (Murphy et al., 2017) (Siemens Healthcare, Germany) on day 21, and the experiment was concluded after 35 days.

#### 2.14.1 Preoperative management

The animals underwent an 8-hour fasting period, with water withheld for 4 h prior to surgery. Intramuscular injections of suxamethone (5 mg/kg) and atropine sulfate (0.08 mg/kg) were administered. They were positioned laterally and monitored until fully anesthetized. An auricular vein catheter was inserted for the administration of lactated Ringer’s solution and propofol to induce anesthesia. Physiological parameters were monitored using limb lead electrocardiography, invasive arterial pressure obtained through femoral artery catheterization, and pulse oximetry. Preoperatively, aspirin (200 mg) was administered.

#### 2.14.2 Surgical procedure

**FIGURE 1:**
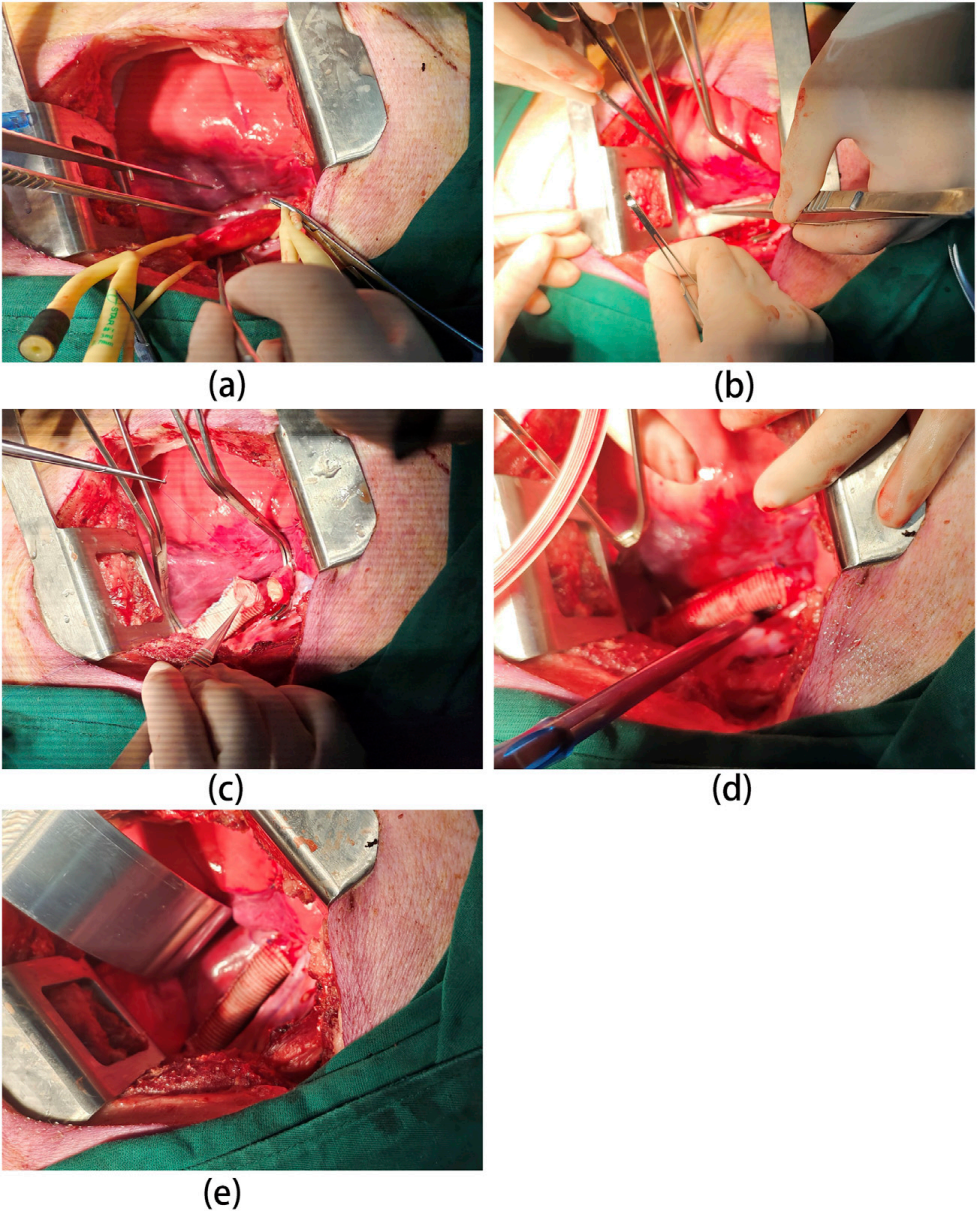
Overview of the surgical procedure. **(A)** Abdominal aorta dissection: porcine abdominal aorta is carefully dissected to expose the area requiring replacement. This may involve mobilizing surrounding tissues and minimizing blood vessel injury. **(B)** Proximal Anastomosis: the proximal end of the vascular graft (UPCCVG) is anastomosed (sutured) to the native aorta in an end-to-side fashion. This ensures proper blood flow continuation beyond the replaced segment. **(C)** Distal Anastomosis: the distal end of the vascular graft is then anastomosed to the remaining healthy portion of the abdominal aorta. This completes the graft interposition within the aortic flow path. **(D)** Graft Patency Confirmation: After anastomosis completion, the vascular graft is opened, allowing blood flow to re-establish through the graft. Careful observation and hemodynamic monitoring confirm proper graft function. **(E)** Surgical Field Closure: Once hemostasis (bleeding control) is achieved and graft patency verified, the surgical field is meticulously closed in layers to minimize complications.

The surgical procedure was shown in Figures 1A–E. A midline laparotomy of approximately 10 cm was performed to expose the abdominal aorta. Following aortic clamping, heparin (200 U/kg) was administered intravenously for anticoagulation. The aorta was transected between undamaged proximal and distal segments, with a 4 cm segment resected. The vascular graft was interposed and end-to-end anastomosed to the aortic segments using an 8-0 polypropylene suture. The muscle and skin layers were closed with 2-0 and 4-0 Vicryl sutures, respectively. After confirming graft patency by ultrasound, anesthesia was reversed, and the animals recovered in their cages.

#### 2.14.3 Post-operative care and graft explantation

Postoperatively, a combination antiplatelet therapy regimen was administered, consisting of aspirin (100 mg/day) and dipyridamole (75 mg/day) for 3 days. At 5 weeks postoperatively, the animals underwent graft explantation. Under general anesthesia, a midline laparotomy was performed through the original incision site. The abdominal cavity was accessed, and the abdominal aorta in the surgical field was carefully dissected to visualize the implanted graft and surrounding tissues. The explanted graft segment was then isolated along with a 4 cm section of the native aorta on either side, ensuring complete graft inclusion. The excised segment was meticulously rinsed with normal saline solution to remove residual blood and subsequently fixed in formalin solution for further analysis.

### 2.15 Histological analysis

Tissue specimens were fixed in 4% formalin solution and subsequently embedded in paraffin. Eight-micrometer-thick sections were deparaffinized through three 5-minute washes in xylene each. To rehydrate the sections, they were immersed in a descending ethanol series (99.9%, 95%, and 70%) followed by rinses in double-distilled water (ddH_2_O). Staining protocols including Hematoxylin and eosin (HE), Masson’s Trichrome (MT), and Verhoeff-Van Gieson (VVG) were conducted following the manufacturer’s instructions. After staining, the sections were dehydrated in an ascending ethanol series (50%, 70%, 80%, 90%, and 100%, with two changes for each concentration lasting 15–20 min) and then mounted onto slides for microscopic examination.

### 2.16 Immunohistochemical analysis

Immunohistochemical analysis was performed to characterize the cellular composition of the implanted vascular graft specimen. Prior to antibody incubation, the sections were blocked in 5% bovine serum albumin in PBS for 30 min at room temperature. Subsequently, they were incubated with specific primary antibodies overnight at 4°C. Smooth muscle cells were labeled using anti-α-SMA antibody (1 μg/mL; Abcam, ab7817), while endothelial cells were identified using anti-VWF antibody (1:200; Abcam, ab11713). Following primary antibody incubation, sections were sequentially incubated with appropriate biotinylated secondary antibodies (1:1,500, Vector) and streptavidin-horseradish peroxidase (Vector). Prior to dehydration and cover slipping, all samples were counterstained with Gill’s hematoxylin (Vector).

### 2.17 SEM morphological analysis of UPCCVG before and after implantation

After dissection, samples were rinsed with saline and then fixed in 4% glutaraldehyde for 4 h. Subsequently, samples were dehydrated through a graded series of ethanol solutions with increasing concentrations (50%, 70%, 80%, 90%, 100%) for two washes lasting 15–20 min each at room temperature. Following dehydration, samples were rinsed in phosphate-buffered saline and incubated overnight, followed by five additional rinses with PBS. Dehydration was followed by vacuum desiccation at room temperature for 8 h. Before imaging, samples were sputter-coated with gold and observed using a SEM (Zeiss Gemini300, 15 kV, 10 µA emission current).

### 2.18 Statistical analysis

The measured results were presented as the arithmetic mean and standard deviation of three parallel patterns. Statistical analysis was conducted using one-way analysis of variance (ANOVA) followed by Tukey’s post-hoc test, performed using SPSS statistical analysis software (ver. 23.0, IBM Corp, Armonk, NY, United States). Statistical analysis was conducted at a confidence level of 95% (*p* < 0.05).

## 3 Results and discussion

### 3.1 Characterization of uncrosslinked porcine collagen for coating

**FIGURE 2:**
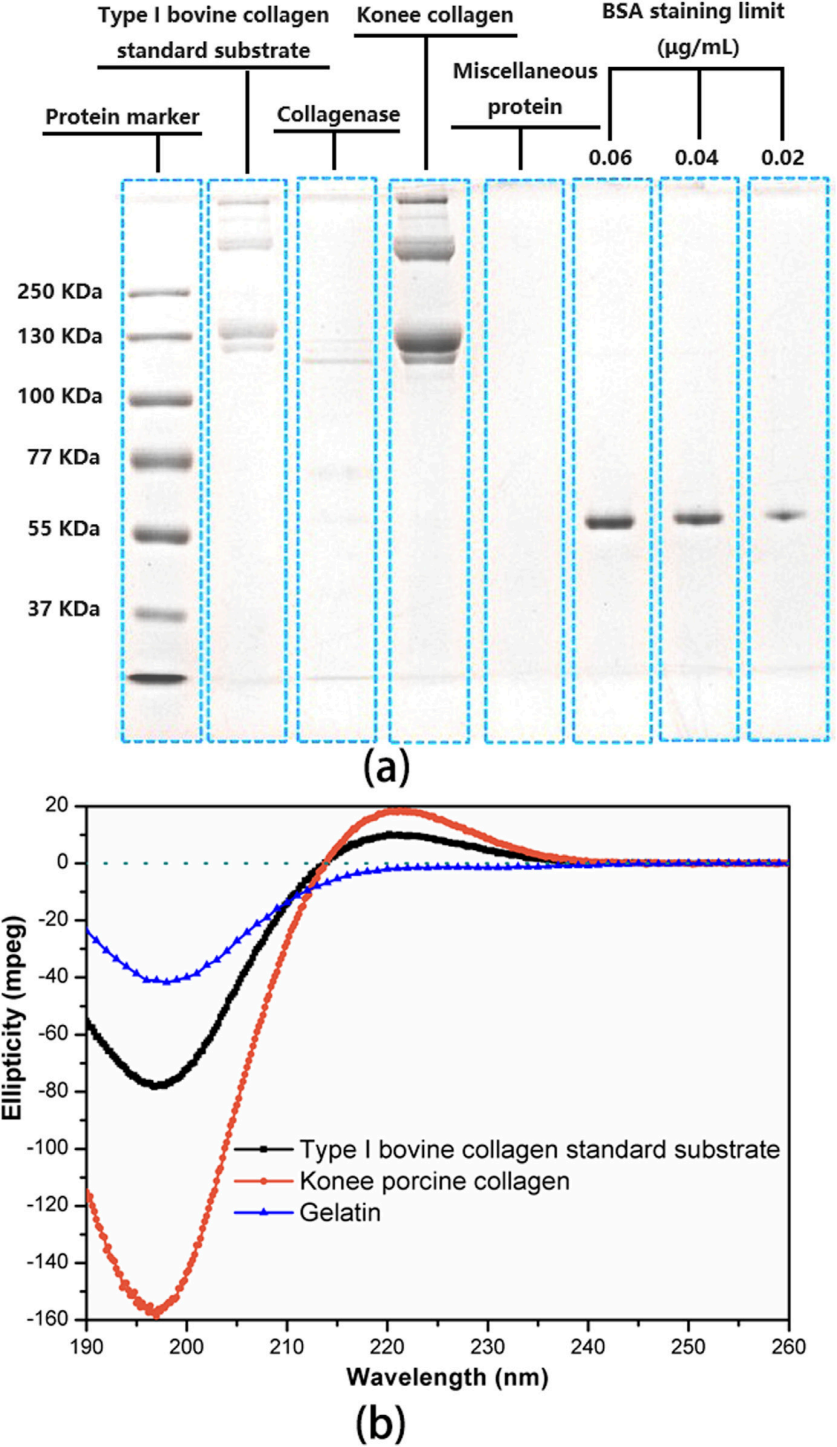
Characterization of uncrosslinked porcine collagen for coating. **(A)** SDS-PAGE analysis of uncrosslinked porcine collagen, protein markers, and type I bovine collagen standard substrate. The limit staining concentration of BSA in the protein markers was determined to be 0.02 μg/mL. ImageJ software was employed to quantify the band intensity of each lane for subsequent collagen purity calculations. **(B)** CD spectrum of uncrosslinked porcine collagen, type I bovine collagen standard substrate with complete triple helix structure and gelatin without triple helix structure introduced as controls.

The molecular weight of the porcine collagen utilized for coating was determined through SDS-PAGE analysis, as depicted in Figure 2A. The bands of Konee porcine collagen matched those of the type I bovine collagen standard substrate, displaying two α bands (α1 and α2), a dimer β band, and a trimer γ band. The α1 band exhibits twice the strength of the α2 band, with the α1 chain weighing approximately 130 KDa, slightly higher than the α2 chain (Song et al., 2019; Xiao et al., 2023). Both the dimer β band and the trimer γ band have molecular weights exceeding 250 KDa, with a higher β chain content suggesting increased intermolecular crosslinking. The presence of the γ band suggests intramolecular crosslinking among the three chains of the collagen molecule. The electrophoretic bands appeared clear, with the absence of low molecular weight bands, indicating the preservation of collagen’s molecular structure during extraction.

Collagen purity was assessed utilizing ImageJ software to quantify the optical density of protein bands on a gel. Konee porcine collagen, collagenase-digested collagen, collagenase, miscellaneous protein, and type I bovine collagen standard substrate were loaded alongside a BSA-stained band at a limiting concentration of 0.02 μg/mL. Analysis of band intensities indicated that the collagen isolate contained over 99.8% collagen, with less than 0.2% impurities of miscellaneous protein. This indicates a high level of purity for the coating collagen.

CD spectroscopy was utilized to characterize the secondary structure of uncrosslinked porcine collagen intended for coating (Figure 2B). Type I bovine collagen standard substrate, possessing a complete triple-helix structure, and gelatin, a proven collagen breakdown product lacking a triple-helix structure, were introduced as references. The CD spectra of Konee porcine collagen and type I bovine collagen displayed a characteristic positive peak near 222 nm in the far-ultraviolet region, indicating a triple-helical conformation (Qi et al., 2015). Furthermore, a negative peak near 196 nm suggested the presence of a random coil structure. In contrast, gelatin exhibits a negative characteristic peak solely at 196 nm, lacking the characteristic peak at 222 nm, indicative of a fully random structure (Gopal et al., 2012). The ratio of the absolute values of these peak heights, known as the Rpn value, serves as an indicator of both the integrity of the triple helix and the intermolecular packing within type I collagen (Zhang et al., 2020). Significantly, the Rpn value of Konee collagen (0.119) fell within the standard range of 0.09–0.15, indicating a high degree of triple-helical integrity.

The triple-helical structure plays a crucial role in determining collagen’s biological activity. Based on the characterization of uncrosslinked porcine collagen for coating provided above, this collagen exhibits high purity, a complete triple-helical conformation, and serves as the structural foundation for its biological functionality. Consequently, this collagen is well-suited for biomedical applications.

### 3.2 Immunogenicity of uncrosslinked porcine collagen

The immunogenicity of xenogeneic material is one of the most important sources of risk for its clinical transformation and application. Therefore, the immunogenicity of the collagen was systematically controlled and evaluated. Firstly, a relatively stable and safe porcine skin source was established, followed by a specific process route to mostly remove/reduce the immunogenic substances, such as the enzymatic hydrolysis and purification process. Secondly, potential immunogenic substances in the collagen were analyzed and examined, including collagen purity, foreign proteins, DNA residual, fat residual, α-Gal residual and total sugar content. The collagen purity and foreign proteins were researched by SDS-PAGE analysis and results showed that the purity of the purified collagen can reach to more than 99% and the content of foreign protein is less than 1% (YY/T0954, 2015). The DNA residual research indicated that the DNA residual is less than 1.5 ng/mg (YY/T1888, 2023), even meeting the standard requirements for injectable biological products. The fat residual study verified that the fat residual is no more than 0.1g/100g (DB44/T2080, 2017), which is far below than the relevant standard requirements for medical collagen products. Lots of reports have shown that the α-Gal is the main target antigen for xenogeneic rejection. The relevant results showed that the number of α-gal residual is less than 5.6 × 10^11^ per milligram, a far cry from the antigen number in porcine acellular dermal matrix, which is 3.62 × 10^14^ per milligram (Shan et al., 2015). The total sugar content research indicated that the ratio of sugar residual to collagen was no more than 1/1,000. All the relevant potential immunogenic substances studies indicated that the residual in the purified collagen complies with the relevant standards or is lower than similar products on the market. Finally, immunotoxicology experiments *in vivo* in animal models were conducted to further verify the immunogenicity of uncrosslinked collagen according to the related requirements of YY/T 16886.20/ISO 10993-20, including inflammation response, hypersensitivity, immunosuppression and immunostimulation tests. Results indicated that the collagen would not cause inflammatory reactions or hypersensitivity reactions and have no effect on the immunological organs, hematological indicators, lymphocyte proliferation, and lymphocyte subset detection. Besides, it would not cause humoral immune response. The immunostimulation produced by pollutants other than the materials is very low, and the impact on humoral immune function, cellular immunity and transformation function of mouse T lymphocytes is quite subtle and limited. All these studies indicated that the collagen has almost no immunotoxicity.

### 3.3 Optimizing coating composition for UPCCVG

**FIGURE 3:**
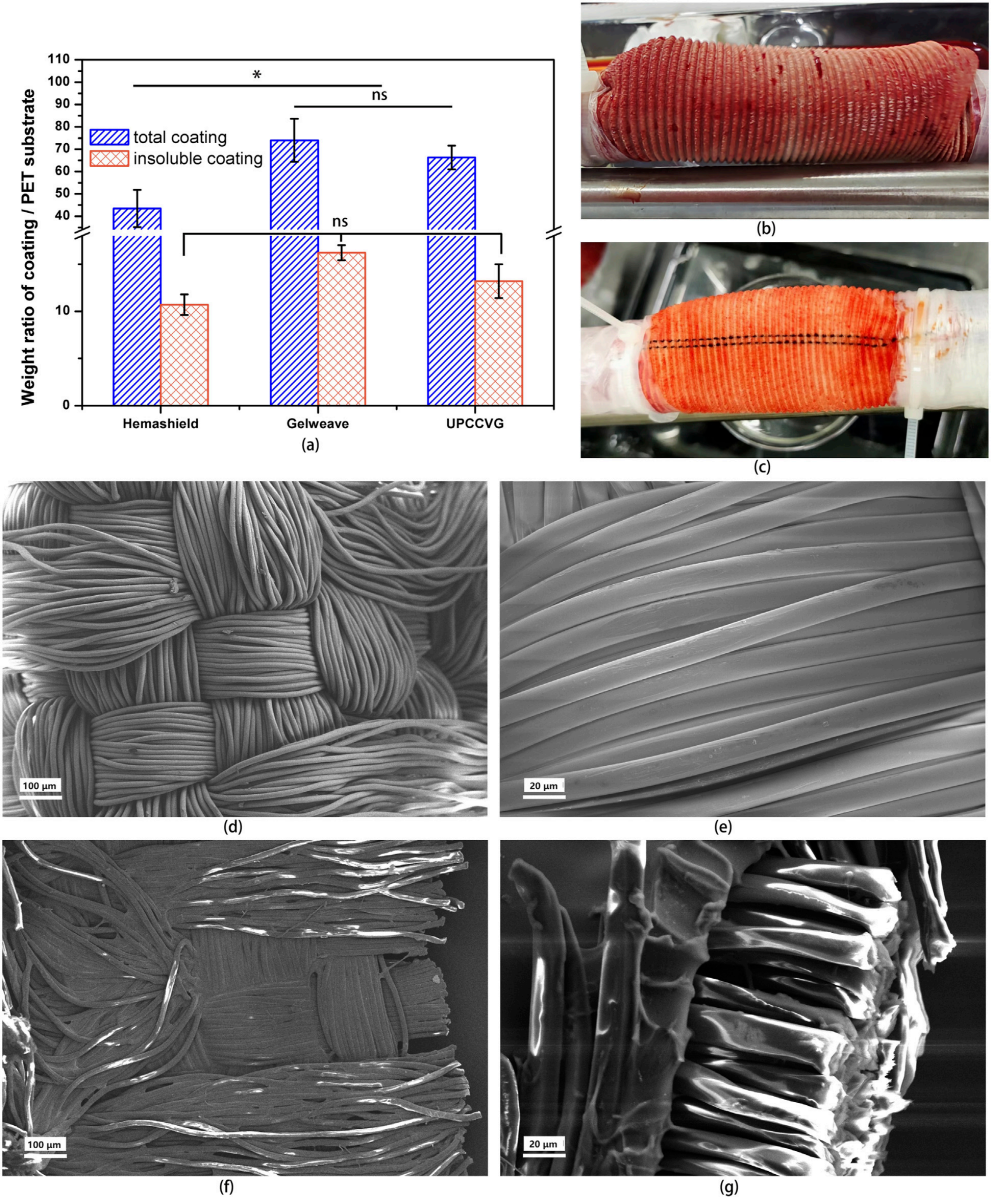
Performance Characterization of UPCCVG. **(A)** Coating/PET substrate weight ratio analysis of UPCCVG compared to commercially available vascular grafts. The total coating weight ratio (including both water-insoluble and water-soluble components) of UPCCVG is not significantly different from Gelweave (*p* > 0.05), but both are significantly different from Hemashield (*p* < 0.05). No significant difference was observed in the weight ratio of water-insoluble coatings between the three groups (*p* > 0.05). **(B, C)** Blood Leakage resistance of UPCCVG with different coating content during extracorporeal circulation. **(D–G)** SEM images of inner wall morphology of PET substrate without coating [**(D)** ×100; **(E)** ×500] and UPCCVG with coating [**(F)** ×100] as well as cross section [**(G)** ×500].

An optimal coating for vascular grafts should fill the fibrous gaps within the PET substrate while promoting subsequent cellular growth. This study investigated the coating ratio of UPCCVG and compared it with those of commercially available vascular grafts. A two-step extraction process was employed to quantify collagen content. Initially, water-soluble components such as plasticizer glycerol and small collagen fragments (degraded during radiation sterilization) were extracted with purified water. Subsequently, water-insoluble components, including intact collagen molecules and a minor portion of crosslinked collagen formed during sterilization, were extracted with collagenase solution. Sufficient collagenase can fully degrade the crosslinked gelatin coating as well. Figure 3A illustrates the proportions of coating components in UPCCVG and the two commercial vascular grafts.

Hemashield, a representative commercial graft, employs a mildly crosslinked collagen/glycerol coating with a total ratio of 43.5% ± 8.3%, aligning with previous literature (Machy et al., 2002). Gelweave, on the other hand, utilizes a highly crosslinked gelatin/glycerin coating, resulting in a total ratio of 74% ± 9.6%. The UPCCVG coating ratio was 66.3% ± 5.3%. Statistical analysis showed no significant difference between the coating ratios of UPCCVG and Gelweave (*p* > 0.05), but a significant difference was observed between both and Hemashield (*p* < 0.05). Interestingly, no significant difference was found in the insoluble coating ratios of Hemashield (10.7% ± 1.1%), Gelweave (16.2% ± 0.8%), and UPCCVG (13.2% ± 1.8%).

Due to collagen’s high molecular weight (approximately 300 KDa) and its inherent insolubility in water and blood, a lower coating ratio is adequate to fill PET substrate gaps when mild crosslinking is applied. In contrast, gelatin has a considerably lower molecular weight and is highly soluble in water and blood. Thus, effective closure of PET substrate gaps with gelatin requires a higher degree of crosslinking or an increase in the overall coating weight. The uncrosslinked porcine collagen/glycerol coating in this study filled the gaps at a slightly higher weight ratio compared to Hemashield. This can be attributed to the absence of crosslinking between collagen molecules, requiring more coating material for equivalent functionality. In conclusion, uncrosslinked collagen-coated grafts require a greater amount of coating material compared to commercially available grafts coated with crosslinked collagen to achieve similar effects.

### 3.4 SEM morphology study of UPCCVG

SEM was utilized to characterize the surface and cross-sectional morphology of UPCCVG and compared it with that of PET substrate without coating (Figures 3D–G). The PET substrate (Figures 3D, E) displayed a fiber tube structure consisting of interwoven PET yarn strands. Following collagen coating, a distinct layer became evident on the surface of the PET substrate fibers (Figure 3F). Additionally, the PET substrate of vascular grafts typically develops wavy crimps along the axial length through a heat treatment process, providing it with a degree of elasticity and deformation capability, thereby conferring anti-twisting properties (Singh et al., 2015; Doersam et al., 2022). Notably, cross-sectional analysis (Figure 3G) revealed collagen infiltration not only on the outer fiber surface but also throughout the internal fiber layers, achieving complete coverage. This coating serves two key functions: firstly, it effectively fills gaps between substrate fibers, minimizing potential blood leakage or seepage caused by blood flow impact during implantation. Secondly, the collagen coating offers favorable biological activity, potentially creating a suitable growth environment for implanted cells. SEM observations provide preliminary evidence that the uncrosslinked collagen coating technology has successfully achieved its intended purpose.

### 3.5 Water permeability of UPCCVG

The water permeability of the UPCCVG was assessed and compared with that of PET substrate without coating and commercially available vascular grafts. The PET substrate, composed entirely of yarn bundles with inter-yarn gaps, demonstrated a high initial water permeability of 112.0 ± 9.6 mL·min⁻^1^·cm⁻^2^. However, implantation in this state could lead to excessive bleeding, making it unsuitable for direct clinical application. UPCCVG with uncrosslinked collagen coating significantly reduced water permeability to 6.8 ± 3.3 mL·min⁻^1^·cm⁻^2^, a value within the range observed for established commercial vascular grafts such as Hemashield (11.6 ± 8.0 mL·min⁻^1^·cm⁻^2^) and Gelweave (3.2 ± 1.3 mL·min⁻^1^·cm⁻^2^). The results of statistical analysis for inter-group comparison revealed no significant difference among these three factors (*P* > 0.05). This suggests that UPCCVG possesses water permeability characteristics comparable to clinically approved options.

### 3.6 Evaluation of blood leakage resistance of UPCCVG under extracorporeal circulation

The present study examined the efficacy of uncrosslinked collagen coating in preventing blood leakage from UPCCVG using a prolonged (4 h) extracorporeal circulation model with fresh, anticoagulated porcine blood. This approach aimed to simulate clinical conditions and provide a more rigorous assessment of graft integrity compared to static *in vitro* models. The reference (Madaghiele et al., 2009) used bovine blood as a medium to evaluate the blood retention ability of vascular grafts, which is instructive. The findings revealed a critical dependence of coating content on maintaining a leak-proof graft during circulation. Grafts with minimal collagen coating (e.g., 40% total content) exhibited blood seepage through the wall following irradiation sterilization (Figure 3B). Conversely, grafts with sufficient coating content (e.g., 66.3% total content, as detailed in Figure 3A) effectively prevented blood leakage and seepage throughout the circulation period even after sterilization (Figure 3C), potentially mitigating complications in clinical scenarios. These results highlight the effectiveness of a dynamic porcine blood extracorporeal circulation model for evaluating leakage in uncrosslinked collagen-coated vascular grafts. This method provides a valuable tool for preclinical assessment and optimization of graft design to achieve improved clinical outcomes. It should be noted that the irradiation sterilization process typically has a negative impact on the water permeability and anti-leakage performance of vascular grafts, as confirmed in previous experiments. Therefore, testing samples after irradiation sterilization is necessary to obtain more accurate results.

### 3.7 Radial compliance of UPCCVG

The radial compliance of UPCCVG was slightly higher than that of commercially available collagen-coated vascular grafts (1.89 %± 0.06%/100 mmHg VS 1.83 %± 0.09%/100 mmHg). According to reports, the radial compliance of commercially collagen coated PET-based vascular grafts is between 1.0% and 2.0%/100 mmHg. The coating can slightly increase the radial compliance of vascular grafts, and the radial compliance of vascular grafts with the same material increases slightly with the increase of diameter. The compliance of human thoracic aorta, abdominal aorta and other large diameter aorta is about 5.9%/100 mmHg. The radial compliance of vascular grafts currently used in clinical practice cannot fully match the radial compliance of natural blood vessels, which increases the graft failure rate to a certain extent, which is also an improvement direction for vascular grafts. The radial compliance of UPCCVG in this study is at the same level as that of current commercially vascular grafts, but there is still a gap between the radial compliance of UPCCVG and that of natural blood vessels. Subsequently, the compliance is improved by mixing other materials to further improve the success rate of transplantation.

### 3.8 Blood compatibility of UPCCVG

The results of the blood compatibility test showed that the mean absorbance of UPCCGV was 0.018, and that of commercially collagen coated vascular grafts was 0.019; the hemolysis rate of UPCCVG was very low, only 0.5%, which was like that of commercially collagen coated vascular grafts (0.6%), and far lower than the acceptable limit of 5% hemolysis rate of medical devices. The reason is that the PET substrate and collagen coating in UPCCVG are materials that have been proven for a long time and have good blood compatibility.

*In vivo* thrombosis of UPCCVG was characterized by animal studies from multiple perspectives. During the implantation of UPCCVG, DSA *in vivo* test results of live experimental pigs showed that the internal contour of UPCCVG was smooth, with no visible plaque and stenosis. The inner wall of UPCCVG dissected 5 weeks after implantation was observed, and the inner wall of the vascular graft including the anastomosis was smooth, and no plaque and hematoma were found. Histological analysis of the UPCCVG dissected 5 weeks after implantation showed that there was no significant platelet adhesion and neointima formation in the inner wall of the grafts. Furthermore, SEM was used to observe the inner wall of UPCCVG 5 weeks after implantation, and it was found that the structure of the inner wall tended to transform into natural arterial vessels, and no abnormal blood cells were deposited.

The results of hemolysis test and *in vivo* thrombosis test showed that UPCCVG had good blood compatibility.

### 3.9 Cytotoxicity evaluation of UPCCVG

**FIGURE 4:**
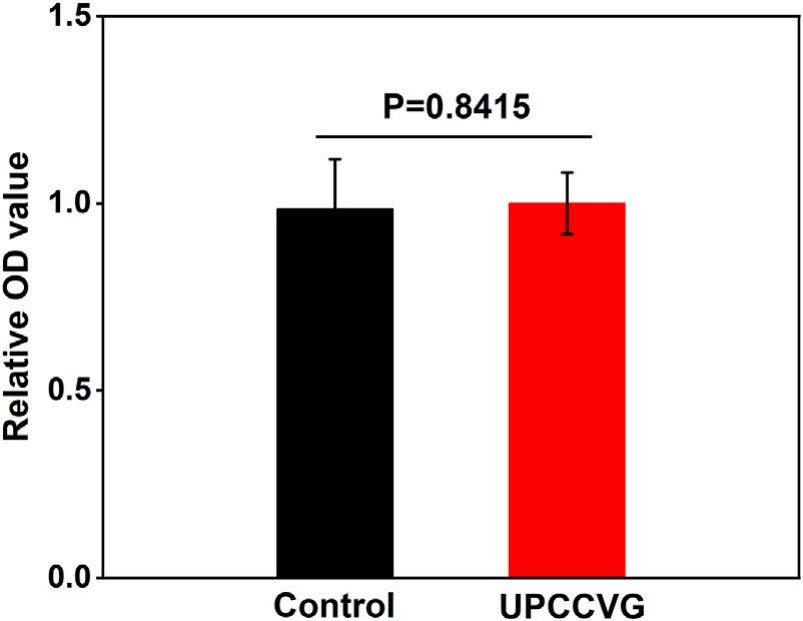
MTT results of UPCCVG extraction solution on MSCs. Normal complete medium was used as control.

MTT assay was used to determine the cytotoxicity of UPCCVG, and results were shown in Figure 4. The results showed that there was no significant difference in cell growth between the control group and the UPCCVG group, indicating no cytotoxicity of the UPCCVG.

### 3.10 Cell proliferation of UPCCVG

**FIGURE 5:**
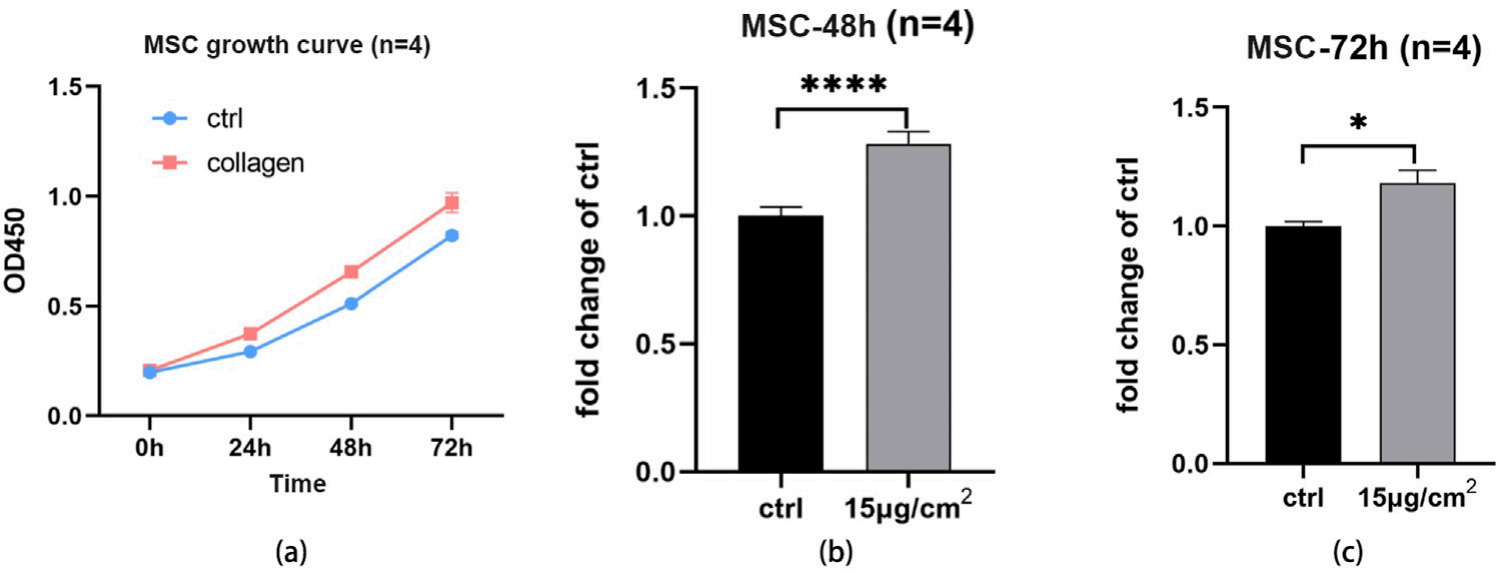
Cell proliferation assay of MSCs on UPCCVG and control surface. **(A)** Growth curve of cells at different times. **(B)** The relative OD value of collagen-coated and control surface at 48 h. **(C)** The relative OD value of collagen coating and control surface at 72 h. n = 3, * indicate *p* < 0.05, *** indicates *p* < 0.001.

The cell proliferation ability of cells on collagen coating of UPCCVG were examined by CCK-8 assay and results were shown in Figure 5. Figure 5A demonstrated that the cells used were in the logarithmic growth phase and suitable for further proliferation assay. After further culture for 48 h and 72 h, the proliferation ability of MSCs on collagen coating was better than that of the control as shown in Figures 5B, C. The collagen coating exhibited good biocompatibility for supporting the cell proliferation.

### 3.11 DSA examination during UPCCVG implantation

**FIGURE 6:**
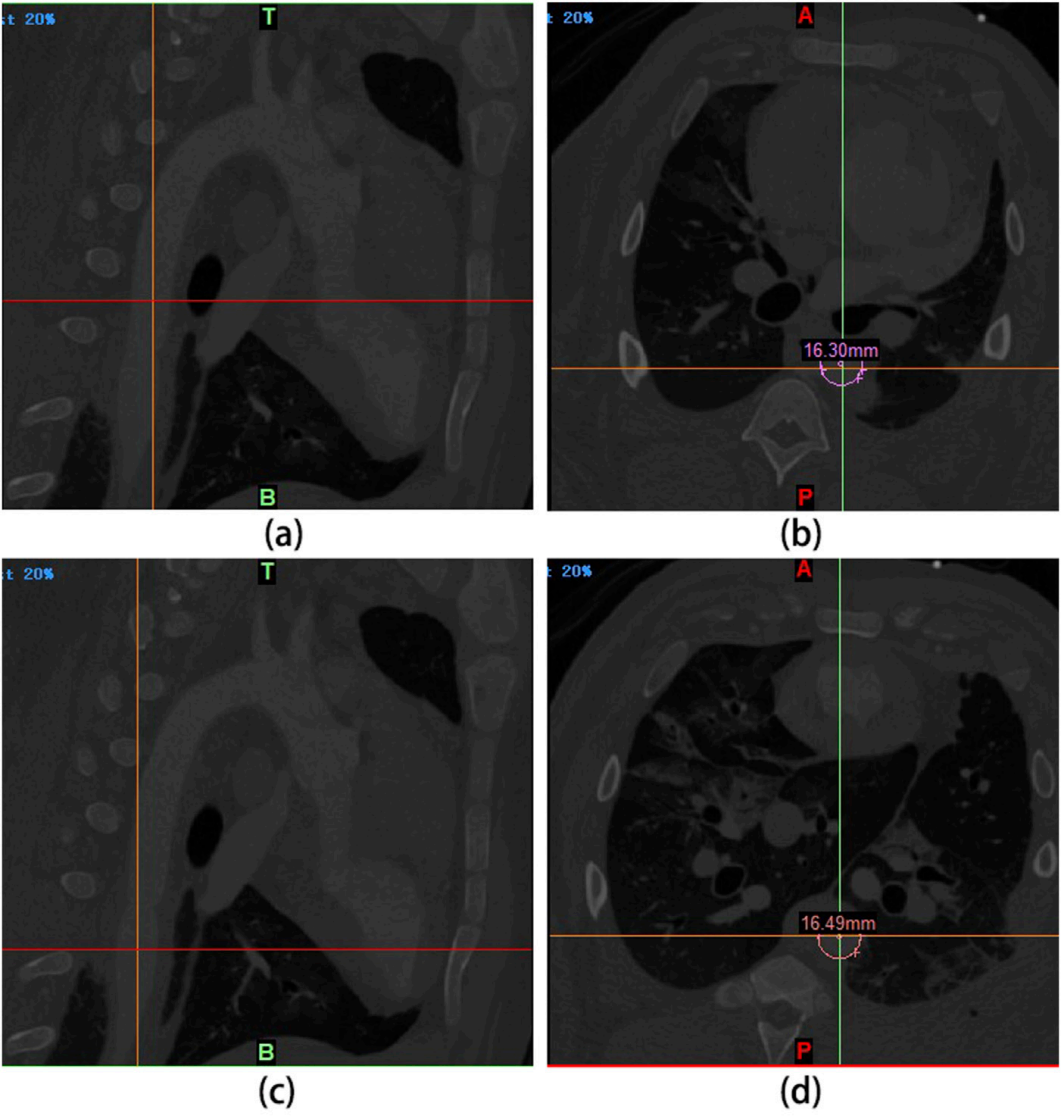
Living conditions of experimental pigs after abdominal aorta replacement and DSA examination results 3 weeks later. **(A, B)**: The intersection of the cursor in **(A)** corresponds to the abdominal aorta proximal to the anastomosis of the vascular graft. The diameter measured at this point is 16.30 mm **(B)**. **(C, D)**: The intersection of the cursor in **(C)** corresponds to the segment of the vascular graft. Its diameter is 16.49 mm, as measured from the corresponding point in **(D)**. These measurements indicate that the graft diameter closely matches the abdominal aorta, with no evidence of stenosis or swelling.

After the implantation of UPCCVG to replace the abdominal aorta, all experimental pigs underwent a postoperative recovery period of approximately 12 h. They demonstrated normal food intake within 24 h post-surgery, and their survival remained uneventful throughout the 5-week observation period. At the 3-week post-implantation mark, DSA examinations were conducted on the pigs (Figure 6). By leveraging digital processing, DSA technology efficiently eliminates superfluous tissue images, retaining solely vascular images. Renowned for its clarity and high resolution, DSA technology offers authentic stereoscopic images for scrutinizing vascular lesions, facilitating the localization, measurement, and diagnosis of vascular stenosis. It serves as an effective modality for evaluating the operational efficacy of vascular grafts (Obara et al., 2020; Ahmed et al., 2022). The DSA findings underscored the patency of the vascular grafts, characterized by a high degree of diameter congruence between the native abdominal aorta (16.30 mm) and the implanted graft (16.49 mm). Multi-sectional DSA scans unveiled no indications of stenosis or graft enlargement. These outcomes collectively affirm the normal functionality of UPCCVG post-implantation.

### 3.12 Anatomic results of UPCCVG after implantation

**FIGURE 7:**
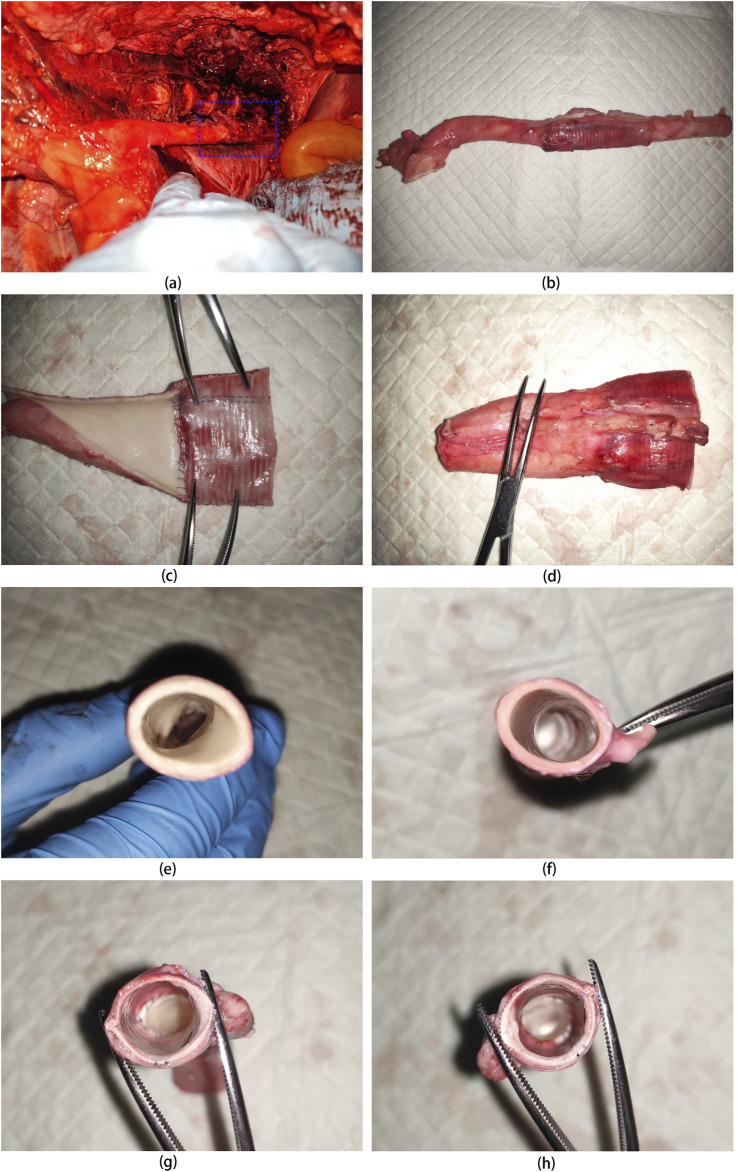
Anatomical Appearance of UPCCVG after 5 Weeks of Pig Abdominal Aorta Replacement. **(A)** Exposed vascular graft 5 weeks postimplantation. **(B)** Vascular graft with both ends anastomosed to the remaining segments of the abdominal aorta. **(C)** Intima of the half section vascular graft connected to the abdominal aorta. **(D)** Outer membrane of the half section vascular graft connected to the abdominal aorta. **(E)** Cross-section of the proximal abdominal aorta. **(F)** Cross-section of the distal abdominal aorta. **(G)** Cross-section of the mid-portion of the vascular graft connected to the proximal abdominal aorta. **(H)** Cross-section of the mid-portion of the vascular graft connected to the distal abdominal aorta.

At 5 weeks post-implantation, the animals were euthanized, and UPCCVG was excised for analysis (Figures 7A–H). Visual inspection revealed an excellent caliber match between the vascular graft and the native artery. The vascular graft appeared fully encapsulated by a neomembrane, demonstrating good integration with surrounding tissues. The UPCCVG graft did not show intraluminal mass formation, thrombosis or stenosis in the lumen, and no intimal hyperplasia appeared at the anastomosis site and inner surface, ensuring smooth blood flow. These observations suggest that the graft is functioning normally and possesses favorable biocompatibility and histocompatibility. This initial evaluation indicates successful graft integration.

### 3.13 Histological analysis

**FIGURE 8:**
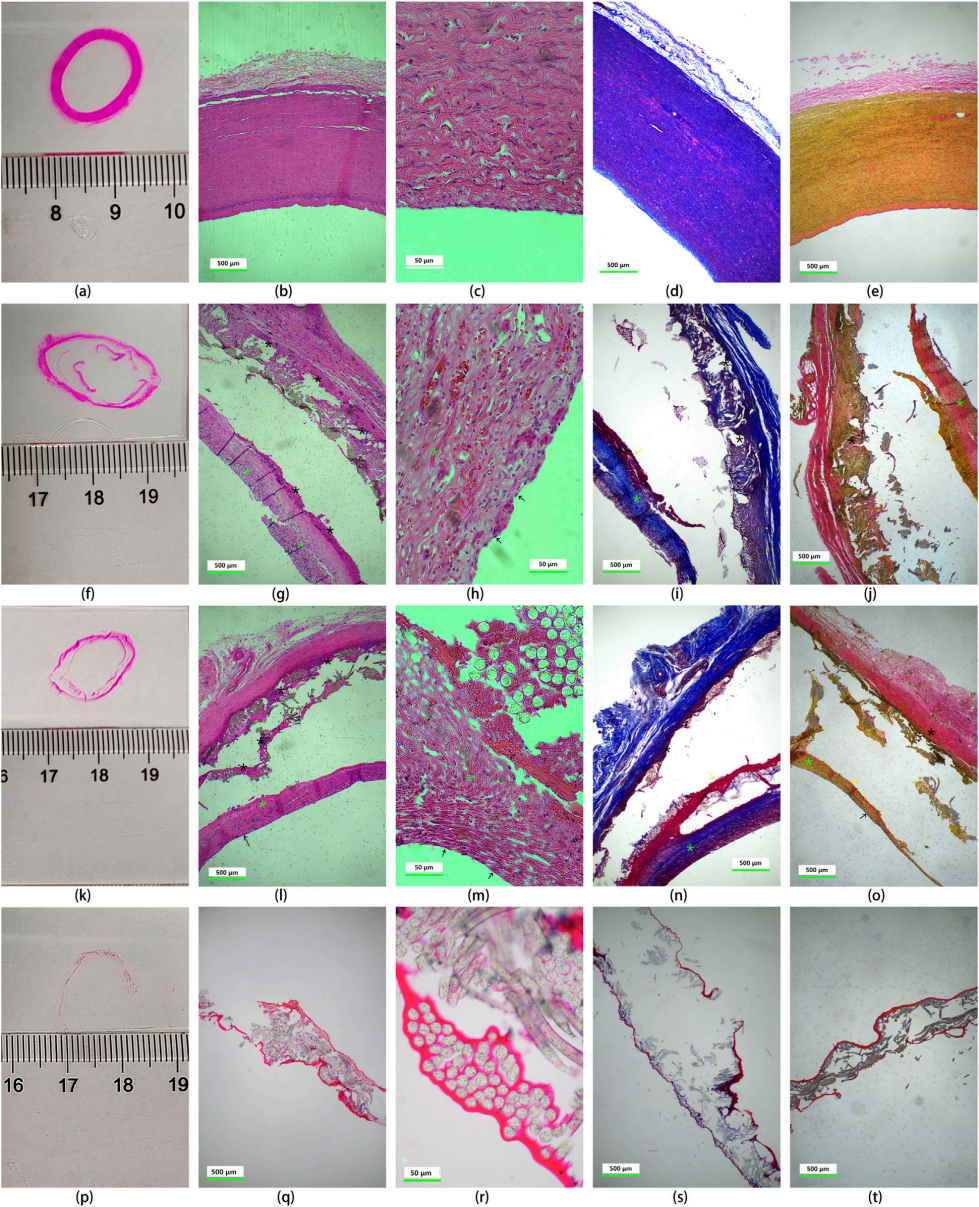
Histological analysis of native arteries and UPCCVG before and after implantation. Rows depict the following conditions: native artery [1st row, **(A-E)**], proximal vascular graft at 5 weeks post-implantation [2nd row, **(F-J)**], middle section of the vascular graft at 5 weeks post-implantation [3rd row, **(K-O)**], and non-implanted, uncrosslinked collagen coated vascular graft [4th row, **(P-T)**]. A schematic diagram of the observation site is presented in 1st column. The subsequent columns display HE staining (2nd column), magnified HE staining (3rd column), Masson’s trichrome staining (4th column), and Verhoeff-Van Gieson staining (5th column). The scale bar of images in 2nd, 4th and 5th columns represents 500 μm; the scale bar of images in 3rd column represents 50 μm. Staining interpretation: HE staining: coated collagen: red (black asterisk); new collagen: pink red (fluorescent green asterisk); Cell nucleus: purple; MT staining: coated collagen: dark red; new collagen: blue; Arterial smooth muscle cells: red; VVG staining: coated collagen: dark red; loose collagen connective tissue: pink; new collagen: yellow; tunica intima: orange. Tissue identification after UPCCVG implantation: black arrow: endothelial cells; yellow arrow: smooth muscle cells.

Explanted UPCCVG were subjected to histological analysis using HE, MT, and VVG staining. The results were compared to those of native arteries and preimplanted grafts (Figure 8). The comparison of tissue staining between native artery and UPCCVG before implantation (Figure 8, 1st row vs. 4th row) revealed that, despite the uncrosslinked collagen coating of UPCCVG being plasticized with glycerol prior to implantation, its staining intensity was significantly lighter than that of natural artery tissue. This suggests that the collagen coating has a denser structure compared to the natural artery. Comparing the tissue staining results before (Figure 8, 4th row) and after (Figure 8, 2nd & 3rd rows) UPCCVG implantation, it was observed that although the collagen coating had not completely degraded after 5 weeks, both the intracavitary and extracavitary surfaces of UPCCVG were covered by and penetrated the new tissue. In Figure 8, HE (g), MT (i and n), and VVG (j and o) staining all demonstrated the presence of residual collagen (dark red staining) within the coating. Previous studies have reported that the complete degradation time of uncrosslinked collagen such as sponge and acellular matrix membrane implanted in animals takes 2–4 months (Gao et al., 2023).

Histological analysis of UPCCVG after implantation (Figure 8, 2nd and 3rd rows) showed that the inner and outer surfaces of the UPCCVG were indeed covered and infiltrated with new tissues. Further analysis showed that these new tissues were extracellular matrix (mainly composed of neocollagen), endothelial cells, and smooth muscle cells. Neocollagen is shown in pink by HE staining, blue by MT staining, and yellow by VVG staining in 2nd and 3rd rows of Figure 8, and is marked by fluorescent green asterisks in the images. The black arrows point to endothelial cells, and the yellow arrows point to smooth muscle cells. The newly formed neointima displayed a similar extracellular matrix composition to native arteries but was significantly different from the preimplanted collagen coating in terms of shape and thickness. HE staining and magnified views revealed an endothelial cell layer thickness of approximately 2.6 ± 0.2 μm, comparable to the reported thickness of human abdominal aortic endothelial cells (around 1 μm) (Krüger-Genge et al., 2019). We chose to compare the results with human abdominal aortic endothelial cells (HAECs) because they are a widely accepted model for understanding endothelial function and responses in a clinical context. While the implant was done in a pig model, which shares many physiological similarities with humans, using HAECs allows us to make more relevant comparisons to potential human clinical outcomes. This approach helps bridge the gap between preclinical animal models and human applications, providing a clearer understanding of how the implant might perform in a human setting.

Interestingly, the explanted grafts displayed a surrounding neotissue layer; however, the adhesion between the neotissue and the graft was weaker than the natural interlayer adhesion within the native artery wall. Paraffin embedding, a common tissue preparation technique, might have contributed to this observation (Soletti et al., 2011). The rapid cooling process inherent to paraffin embedding has been reported to cause partial detachment of neotissue from various materials. Employing frozen sections in future studies could potentially improve the analysis of the neotissue-graft interface (Chaouat et al., 2006; Shi et al., 2019).

### 3.14 Immunohistochemical analysis

**FIGURE 9:**
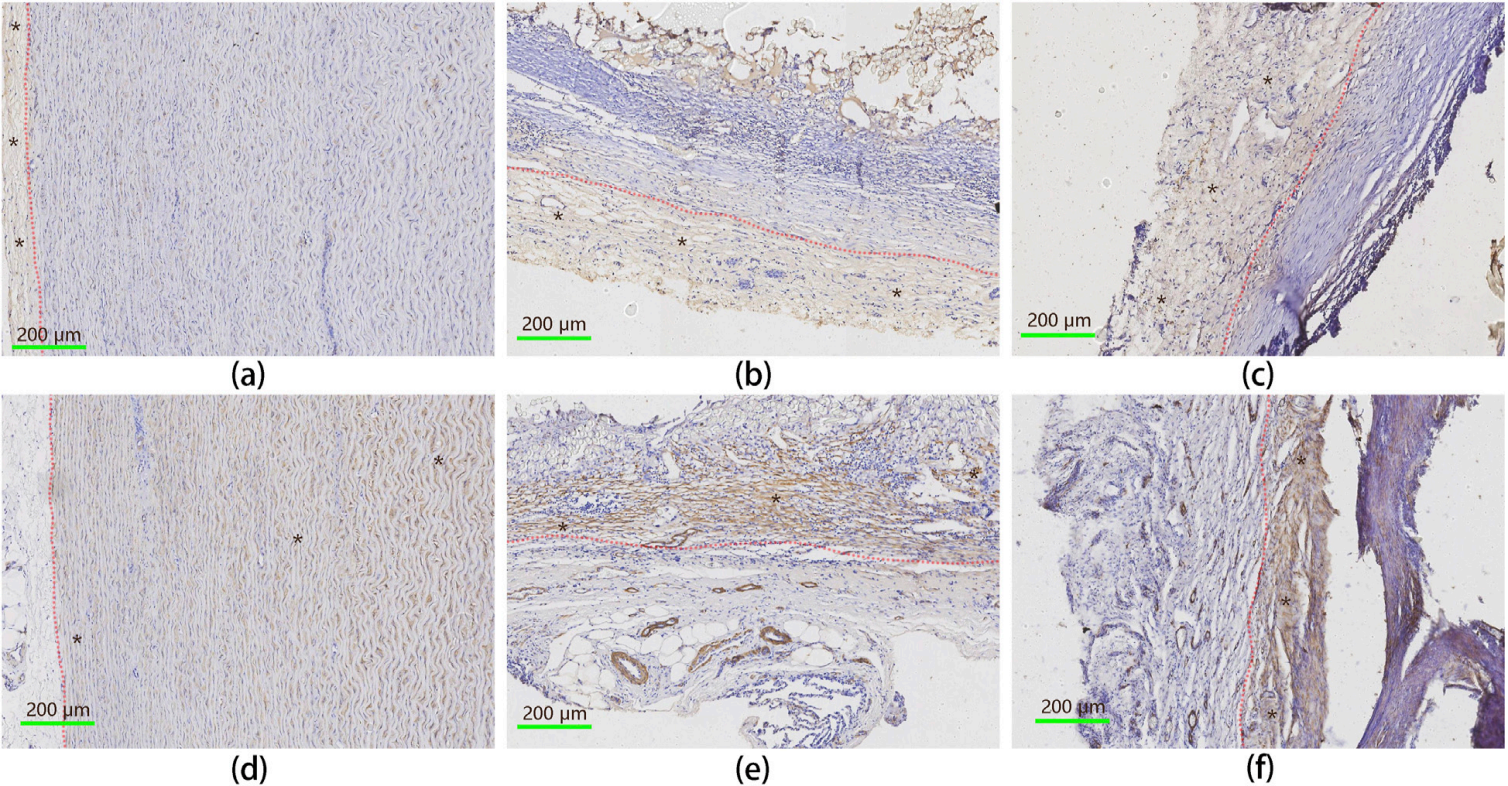
IHC analysis of endothelial and smooth muscle cell distribution in implanted UPCCVG. Top row [**(A–C)**: IHC staining for vWF, identifying endothelial cells. Bottom row **(D–F)**: IHC staining forα-SMA, indicating smooth muscle cells. Left column **(A, D)**: Native artery; Middle column **(B, E)**: Peri-anastomotic region of UPCCVG; Right column **(C, F)**: Middle segment of UPCCVG. Blue-stained particles within the brown-stained regions represent cell nuclei. In the top row, brown staining indicates endothelial cells, while in the bottom row, it corresponds to smooth muscle cells, with black asterisks marking these regions. The native artery displays a well-defined intima layer [**(A)**, brown staining on the left] and a media layer [**(D)**, brown staining on the right], separated by a distinct boundary (indicated by a red dash-dotted line), serving as a positive control. Scale bar = 200 µm.

Immunohistochemistry (IHC) was used to assess the distribution of endothelial cells and smooth muscle cells within the implanted vascular graft, and the results were shown in Figure 9. Von Willebrand Factor (VWF) staining was used as a marker for endothelial cells, while α-Smooth Muscle Actin (α-SMA) identified smooth muscle cells (Skalli et al., 1989; Horvath et al., 2004). The IHC results reveal that the implanted UPCCVG exhibited dense populations of endothelial (Figure 9, upper row) and smooth muscle cells (Figure 9, bottom row) at both anastomosis (Figures 9B, E) and midsection (Figures 9C, F), with relatively clear boundaries. These findings suggest that the inner surface of UPCCVG was successfully endothelialized without intimal hyperplasia, which is typically characterized by smooth muscle cells protruding into the endothelial layer.

### 3.15 SEM morphology study on the internal surface of implanted UPCCVG

**FIGURE 10:**
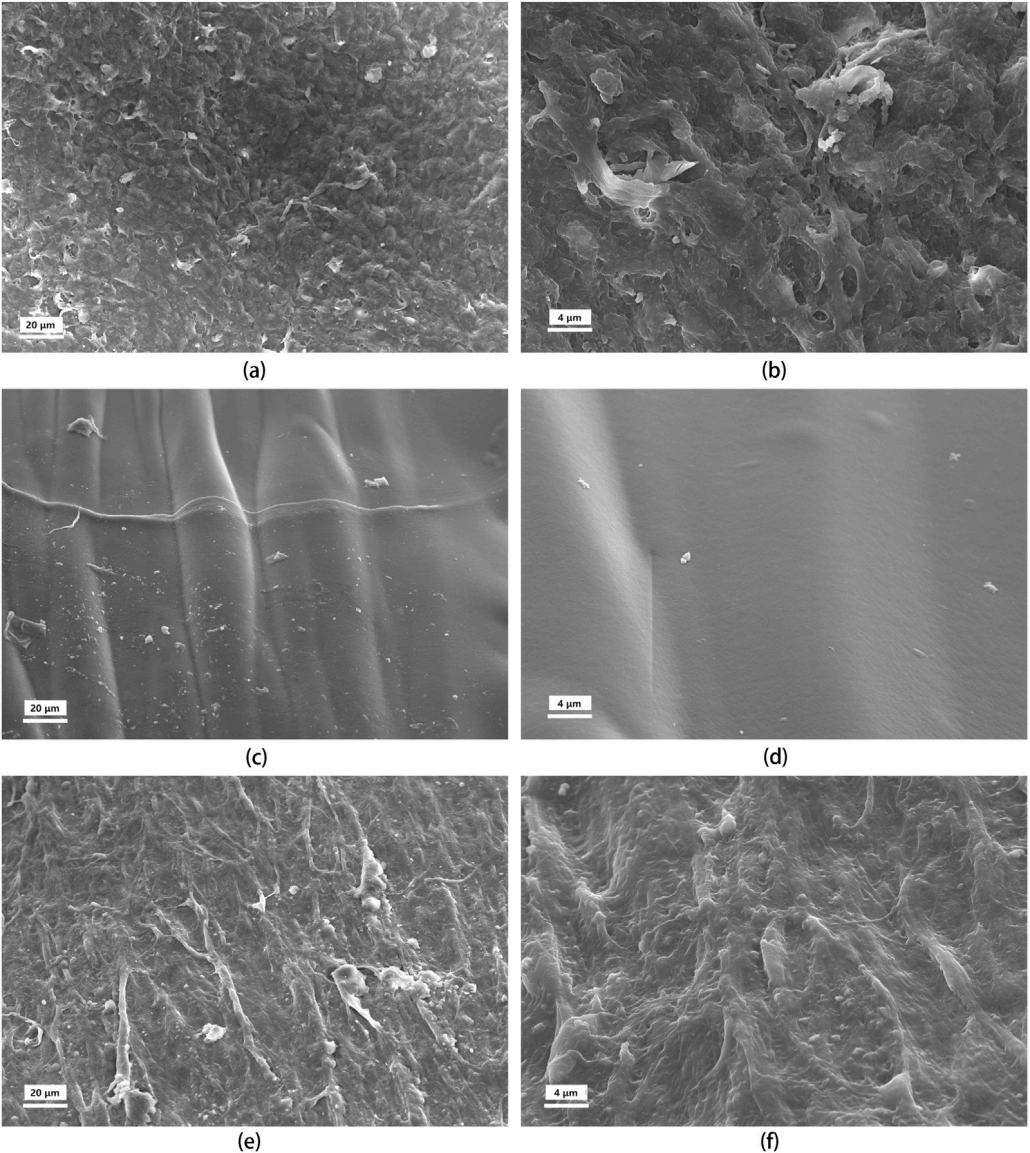
SEM images of the tunica intima of the uncrosslinked collagen coated vascular graft before and after implantation, compared to natural artery. The rows represent the source of the tissue: top row **(A, B)**, natural artery; middle row **(C, D)**, UPCCVG before implantation; bottom row **(E, F)**, UPCCVG after 5 weeks implantation. The columns represent the magnification: left column, ×500; right column, ×2000.

The transformation of the graft’s internal surface was investigated using SEM, comparing its morphology before and after implantation to that of the native artery’s inner wall (Figures 10A–F). The natural artery exhibited a microscopically rough and porous surface, facilitating the exchange of multiple substances. In contrast, the preimplanted graft coating appeared smooth and dense, consistent with the findings from histological staining. Large-molecular-weight dyes infiltrated the artery’s inner and outer layers but struggled to penetrate the preimplanted graft coating, further confirming its denser nature. This initial density is crucial as implanted grafts lack the inherent ability of healthy arteries to adjust tightness under stress, potentially leading to increased fiber gaps.

Remarkably, after 5 weeks *in vivo*, the surface morphology of the neointima transitioned towards mimicking that of the natural artery. The initially smooth surface developed a controlled roughness oriented along blood flow, presumably to minimize shear stress (Krüger-Genge et al., 2019). Additionally, endothelial cells populated the surface of the neointima. These observations demonstrate that while the pre-implantation graft coating differed from the native artery’s inner wall, it progressively transformed post-implantation. This initial densification likely serves a dual purpose: preventing early-stage blood leakage and seepage while promoting neointimal formation and endothelialization in the later stages. In summary, good tissue remodeling occurred on the surface of the vascular graft with uncrosslinked collagen coating, and a fast and good fusion occurred with the surrounding tissue.

### 3.16 Discussion

While crosslinked collagen, gelatin, and albumin-coated grafts have shown some success in clinical settings, a clear academic consensus regarding their precise role in neointima formation and endothelialization remains elusive. This study unveils the potential of UPCCVG as a promising alternative poised to revolutionize existing treatment modalities. Uncrosslinked collagen coatings, compared to their crosslinked counterparts, exhibit superior biosafety and biocompatibility by circumventing the need for additional crosslinking agents and the associated steps of crosslinking agent removal. Under the premise of ensuring no blood leakage during the perioperative period of arterial surgery, the uncrosslinked collagen coating then quickly forms a highly porous structure through natural degradation and turnover processes. This porous structure creates a favorable microenvironment for smooth muscle cells and endothelial cells to infiltrate the graft and stimulate neovascularization (Yan et al., 2019; Niu and Galluzzi, 2021). This ultimately accelerates neointima formation and physiological remodeling, optimizing graft integration and long-term function.

Importantly, this study observed no intimal hyperplasia, a common complication arising from excessive proliferation of smooth muscle cells and excessive precipitation of extracellular matrix within vascular grafts (Jennette and Stone, 2014). The uncrosslinked collagen coating mimics the natural extracellular matrix, promoting host cell adhesion and migration on the graft surface. This enhanced integration reduces inflammation and foreign body response, favoring endothelialization. Endothelialization, the formation of a single endothelial cell layer on the graft lumen, is crucial for maintaining vascular homeostasis and inhibiting smooth muscle cell proliferation, thereby further reducing the risk of intimal hyperplasia (Zhuang et al., 2021). Additionally, endothelialization minimizes thrombosis by regulating blood clotting and platelet adhesion.

In contrast, crosslinked collagen coatings exhibit a denser structure with fewer pores, hindering cell infiltration. This limitation can impede neovascularization by restricting the migration and proliferation of smooth muscle cells and fibroblasts. Consequently, recruitment of endothelial progenitor cells and the action of angiogenic factors may be reduced, compromising neointima formation. Furthermore, the rigidity and slow degradation rate of crosslinked coatings hinder tissue remodeling, potentially leading to poorer graft integration and long-term function. These observations are supported by existing comparative studies on uncrosslinked and crosslinked collagen coating implants. Butler et al. found that in the repair of abdominal hernias with uncrosslinked porcine acellular dermal matrix, host cells were more likely to infiltrate into the interior of the uncrosslinked porcine acellular dermal matrix and had a higher density of neovasculars than in the crosslinked porcine acellular dermal matrix. In addition, better elastic modulus and strength indicate better tissue fusion and remodeling (Butler et al., 2010). Fernandez-Moure et al. showed that compared with the cross-linked acellular dermal matrix, the uncross-linked acellular dermal matrix significantly increased neovascularization during abdominal hernia repair and was more likely to induce neovascularization (Fernandez-Moure et al., 2017). Recent studies have found that vascular substrate stiffness has a significant effect on endothelial and smooth muscle cell autophagy. With the increase of substrate stiffness, endothelial autophagy levels reduced, leading to the reductions in a range of gene expression associated with endothelial function, while VSMCs autophagy level increased, showing a transition from contraction to synthesis (Hu et al., 2021). This study inspires us to consider the influence of surface stiffness of the coating when designing vascular graft coating. It is obvious that the stiffness and modulus of crosslinked coatings are usually higher than that of uncrosslinked coatings, which is not conducive to graft endothelialization.

The stability of vascular grafts was investigated through a series of experiments in this paper. Short-term stability was assessed via *in vitro* blood circulation tests and perioperative observations in animal models. Under pulsed blood flow conditions, the grafts exhibited no leakage, maintained their shape, and showed no abnormal expansion or contraction, indicating good short-term stability. Direct observations during the surgical replacement of pig abdominal aortas confirmed that no blood leakage occurred through the drainage tubes left in the abdominal cavity, and DSA revealed normal graft function during the experimental observation period. Medium-term stability was evaluated through analyses of graft samples 5 weeks post-implantation, which demonstrated normal functioning and solid stability. Long-term stability relies on the graft substrate and the extent of endothelialization. Given that the vascular graft substrate is made of PET fiber—recognized for its durability over decades—the degree of endothelialization significantly impacts stability, with effective endothelialization enhancing graft performance. Our findings indicate that uncrosslinked collagen coating promotes endothelialization, thereby supporting the long-term stability of vascular grafts. However, further investigations are necessary to comprehensively assess the long-term stability of these grafts from multiple perspectives.

This study demonstrates the effectiveness of uncrosslinked pig collagen coating in sealing the PET matrix of vascular grafts, preventing blood leakage during implantation and before neointima formation, thereby mitigating infection risk. Notably, this study confirms the feasibility of UPCCVGs in achieving perioperative hemostasis, followed by neointima formation and endothelialization within an animal model.

However, limitations exist, primarily related to the animal experiment duration and sample size. While the findings are encouraging, further validation with larger cohorts and longer observation periods (4–6 months or more) is necessary. Additionally, parallel animal studies using grafts with crosslinked coatings would provide a more comprehensive comparison of safety and efficacy. In conclusion, this study paves the way for utilizing uncrosslinked collagen coatings in vascular graft development and establishes a solid foundation for future research.

## 4 Conclusion

This study investigated the potential of uncrosslinked collagen as a coating material for vascular grafts. Our findings demonstrate the promise of this approach due to several key advantages:

Biocompatibility and Histocompatibility: Collagen, being the primary component of the extracellular matrix, exhibits excellent biocompatibility and histocompatibility. Uncrosslinked collagen can be completely degraded in the body, and the degradation products are peptides and amino acids, which are benign to the human body. When used as a graft coating, adverse tissue reactions are minimized. Notably, the absence of crosslinking agents further enhances the biosafety profile of this coating material.

*In Vitro* Performance: The *in vitro* evaluation revealed no significant difference between uncrosslinked collagen-coated grafts and commercially available grafts, indicating the feasibility of this coating strategy.

Intima Formation and Endothelialization: Uncrosslinked collagen coatings potentially promote desirable outcomes by facilitating rapid intima formation and subsequent endothelialization.

In conclusion, uncrosslinked collagen presents itself as a promising coating material for vascular grafts. Its biocompatible nature, along with its ability to promote intima formation and endothelialization, warrants further investigation in animal models and potentially future clinical applications.

## Data Availability

The original contributions presented in the study are included in the article/supplementary material, further inquiries can be directed to the corresponding authors.

## Abbreviations

BSA, bovine serum albumin; CD, circular dichroism; CP, Collagen purity; DSA, digital subtraction angiography; HE, Hematoxylin and eosin; IHC, Immunohistochemistry; MT, Masson’s Trichrome; PET, Polyethylene terephthalate; SDS-PAGE, sodium dodecyl sulfate -polyacrylamide gel electrophoresis; SEM, Scanning electron microscope; α-SMA, alpha-smooth muscle actin; UPCCVG, uncrosslinked porcine collagen coated vascular graft; VVG, Verhoeff-Van Gieson; vWF, von Willebrand factor.
